# Supplementary material for: Mechanisms of HSV-1 helicase–primase inhibition and replication fork complex assembly
Source: bioRxiv. 2025 Dec 23:2025.12.23.696259. Preprint. [Version 1] doi: 10.64898/2025.12.23.696259 (PMC12776037; doi:10.64898/2025.12.23.696259)
Supplement: Supplement 1 [file NIHPP2025.12.23.696259v1-supplement-1.pdf]

bioRxiv preprint doi: https://doi.org/10.64898/2025.12.23.696259; this version posted December 24, 2025. The copyright holder for this preprint (which was not certified by peer review) is the author/funder, who has granted bioRxiv a license to display the preprint in perpetuity. It is made available under aCC-BY 4.0 International license.

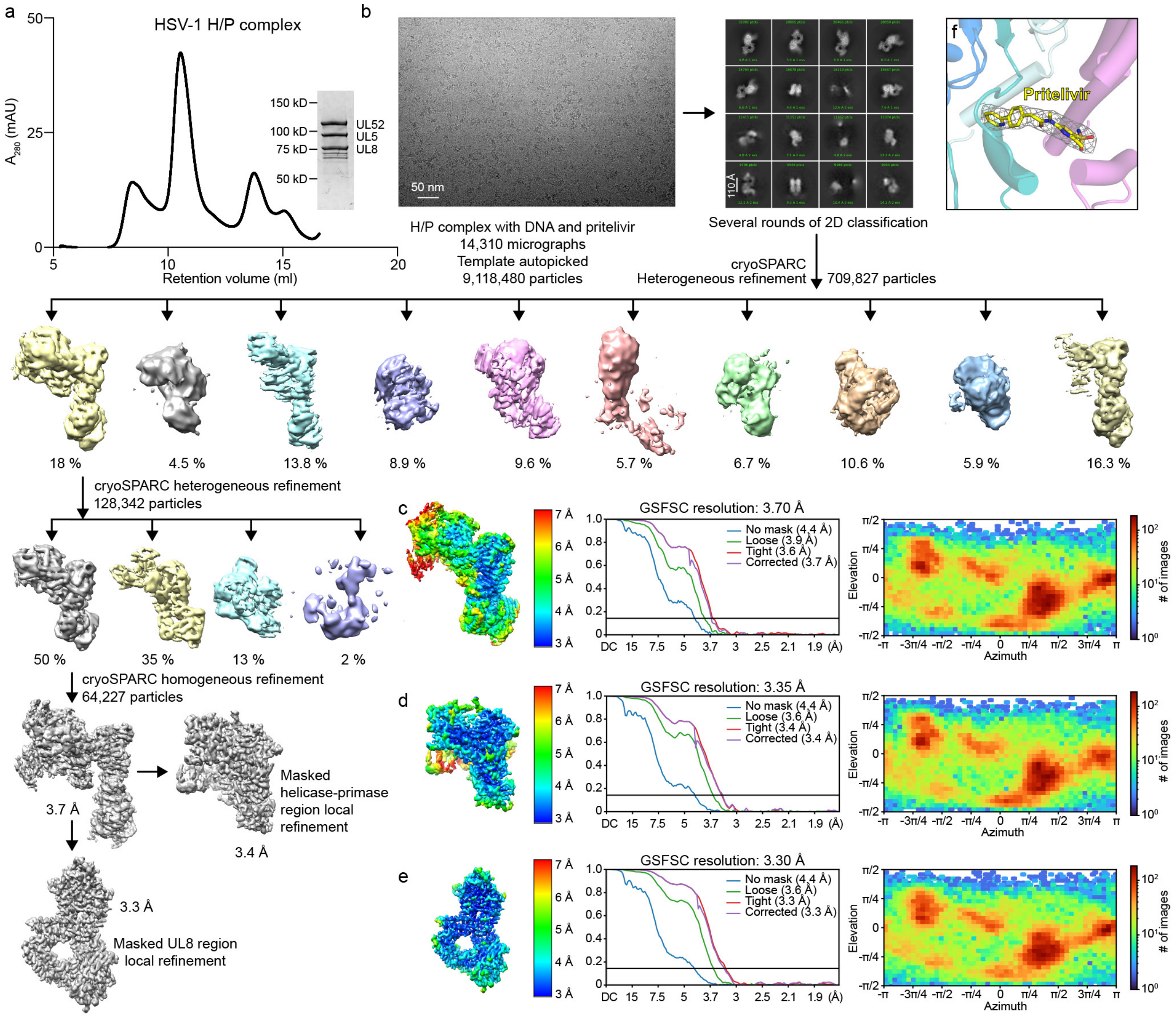

Figure S1. Structure determination of the pritelivir-bound HSV H/P complex.

(a) Size-exclusion chromatography profile and SDS-PAGE analysis of the HSV H/P complex using a Superdex 200 Increase column. The retention volume of the main peak is indicated for each chromatogram. The bands that are smaller than UL8 likely represent degradation products or contaminants.

(b) Workflow used for cryo-EM data processing of pritelivir-bound HSV H/P complex.

(c–e) Local resolution estimation, Fourier shell correlation (FSC) curves, and particle angular distributions of the cryo-EM reconstructions of the overall pritelivir-bound HSV H/P complex at 3.7 Å resolution (c), masked helicase-primase region at 3.4 Å resolution (d) and masked UL8 region at 3.3 Å resolution (e).

(f) Cryo-EM density of pritelivir in the drug binding site of the HSV H/P complex. Pritelivir is shown as sticks.

bioRxiv preprint doi: https://doi.org/10.64898/2025.12.23.696259; this version posted December 24, 2025. The copyright holder for this preprint (which was not certified by peer review) is the author/funder, who has granted bioRxiv a license to display the preprint in perpetuity. It is made available under aCC-BY 4.0 International license.

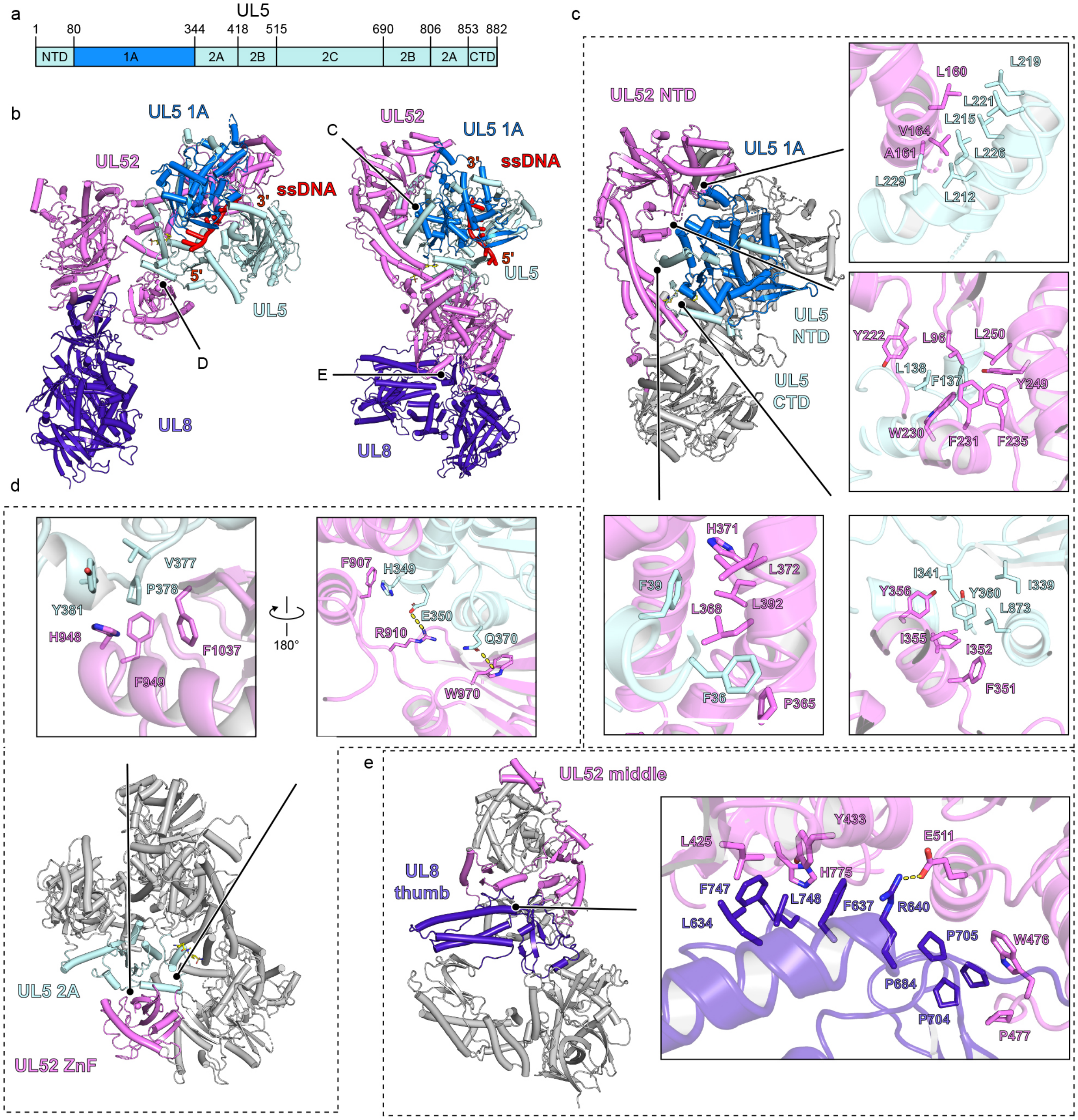

Figure S2. Domain architecture of the HSV H/P complex.

(a) Schematic diagram of the domain architecture of the HSV helicase UL5 and primase UL52.

(b) Ribbon diagram of the HSV H/P complex bound to ssDNA and pritelivir.

(c) Interactions between the primase UL52 NTD and UL5 NTD, 1A domain, and CTD.

(d) Interactions between the primase UL52 ZnF domain and UL5 2A domain.

(e) Interactions between the primase UL52 middle domain and UL8 thumb domain.

bioRxiv preprint doi: https://doi.org/10.64898/2025.12.23.696259; this version posted December 24, 2025. The copyright holder for this preprint (which was not certified by peer review) is the author/funder, who has granted bioRxiv a license to display the preprint in perpetuity. It is made available under aCC-BY 4.0 International license.

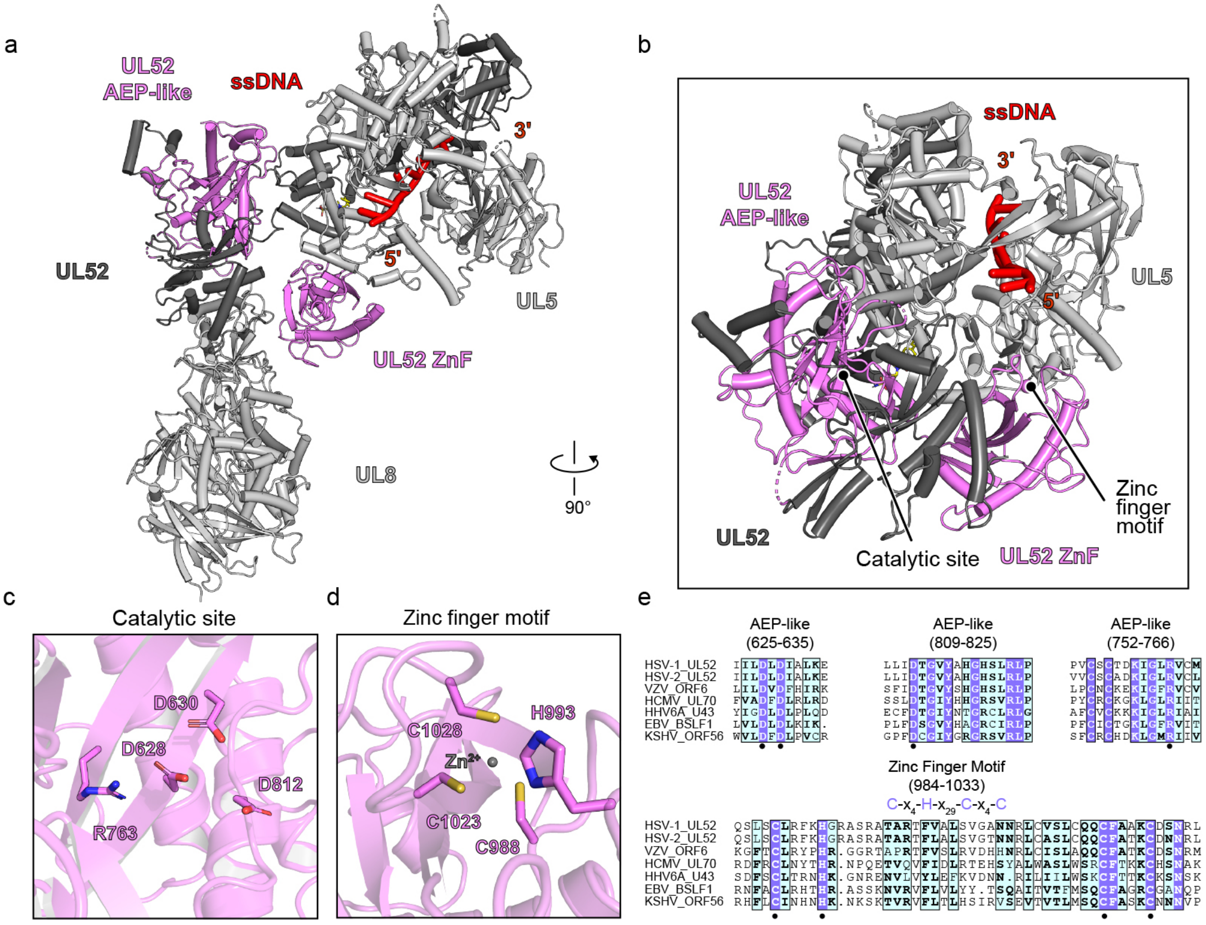

Figure S3. The HSV H/P complex primase active site.

(a) Ribbon diagram of the HSV H/P complex bound to ssDNA and pritelivir. The AEP-like and ZnF domains in UL52 are labeled.

(b) Close-up view of the UL52 AEP-like and ZnF domains. The locations of the AEP-like active site and zinc finger motifs are indicated.

(c) HSV primase UL52 catalytic site in the AEP-like domain.

(d) HSV primase UL52 zinc binding site in the ZnF domain. A zinc ion is shown as a sphere.

(e) Sequence alignment of herpesvirus primase active sites and zinc finger motifs. Conserved active site residues are indicated. HSV-1, herpes simplex virus 1; HSV-2, herpes simplex virus 2; VZV, varicella-zoster virus; HCMV, human cytomegalovirus; EBV, Epstein–Barr virus; HHV-6A, human herpesvirus 6A. KSHV, Kaposi's sarcoma-associated herpesvirus.

bioRxiv preprint doi: https://doi.org/10.64898/2025.12.23.696259; this version posted December 24, 2025. The copyright holder for this preprint (which was not certified by peer review) is the author/funder, who has granted bioRxiv a license to display the preprint in perpetuity. It is made available under aCC-BY 4.0 International license.

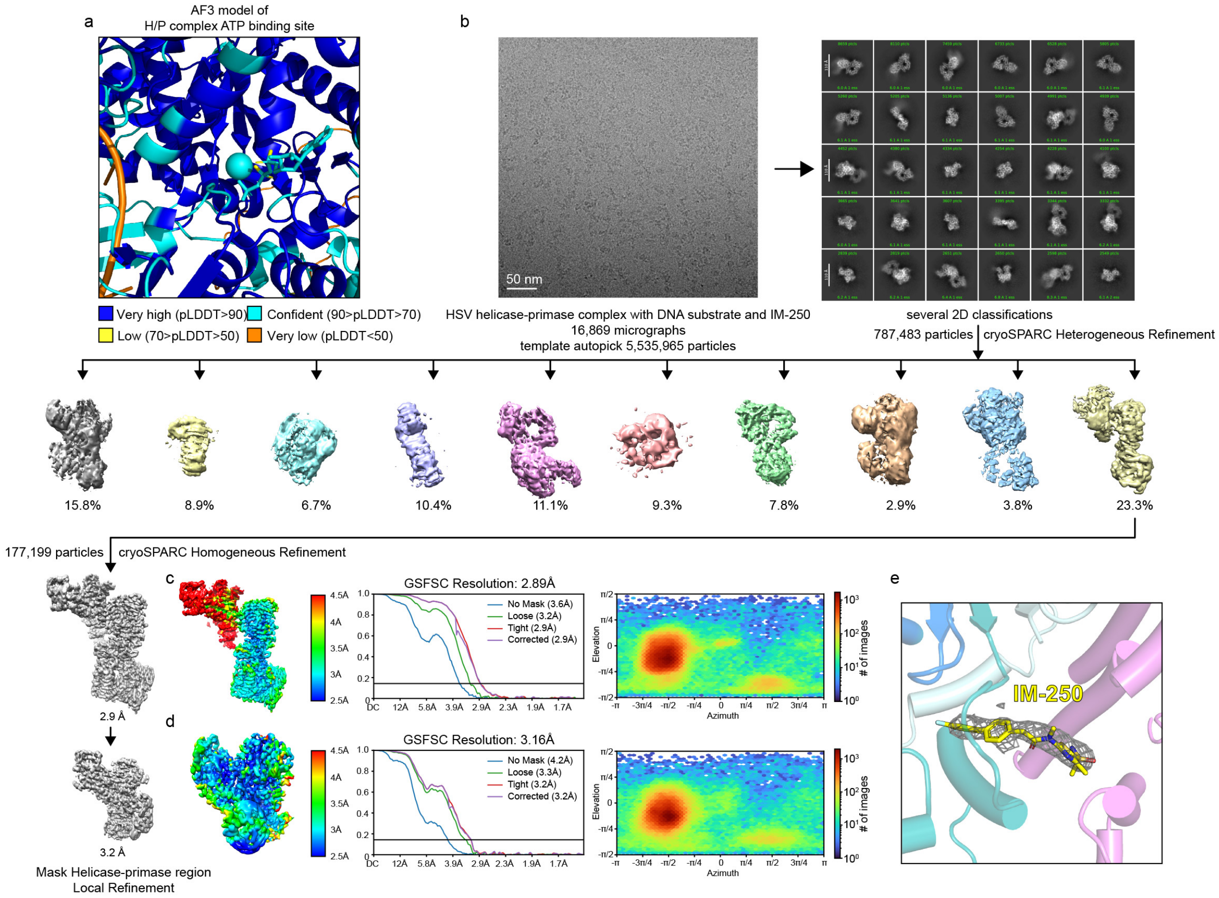

Figure S4. Structure determination of the IM-250 bound HSV H/P complex.

(a) pLDDT of the ATP binding site of the AF3 model of ATP-bound HSV H/P complex.

(b) Workflow used for cryo-EM data processing of IM-250-bound HSV H/P complex.

(c and d) Local resolution estimation, Fourier shell correlation (FSC) curves, and particle angular distributions of the cryo-EM reconstructions of the IM-250-bound HSV H/P complex at 2.9 Å resolution (c) and masked helicase–-primase region at 3.2 Å resolution (d).

(e) Cryo-EM density of IM-250 in the drug binding site of the HSV H/P complex. IM-250 is shown as sticks.

bioRxiv preprint doi: https://doi.org/10.64898/2025.12.23.696259; this version posted December 24, 2025. The copyright holder for this preprint (which was not certified by peer review) is the author/funder, who has granted bioRxiv a license to display the preprint in perpetuity. It is made available under aCC-BY 4.0 International license.

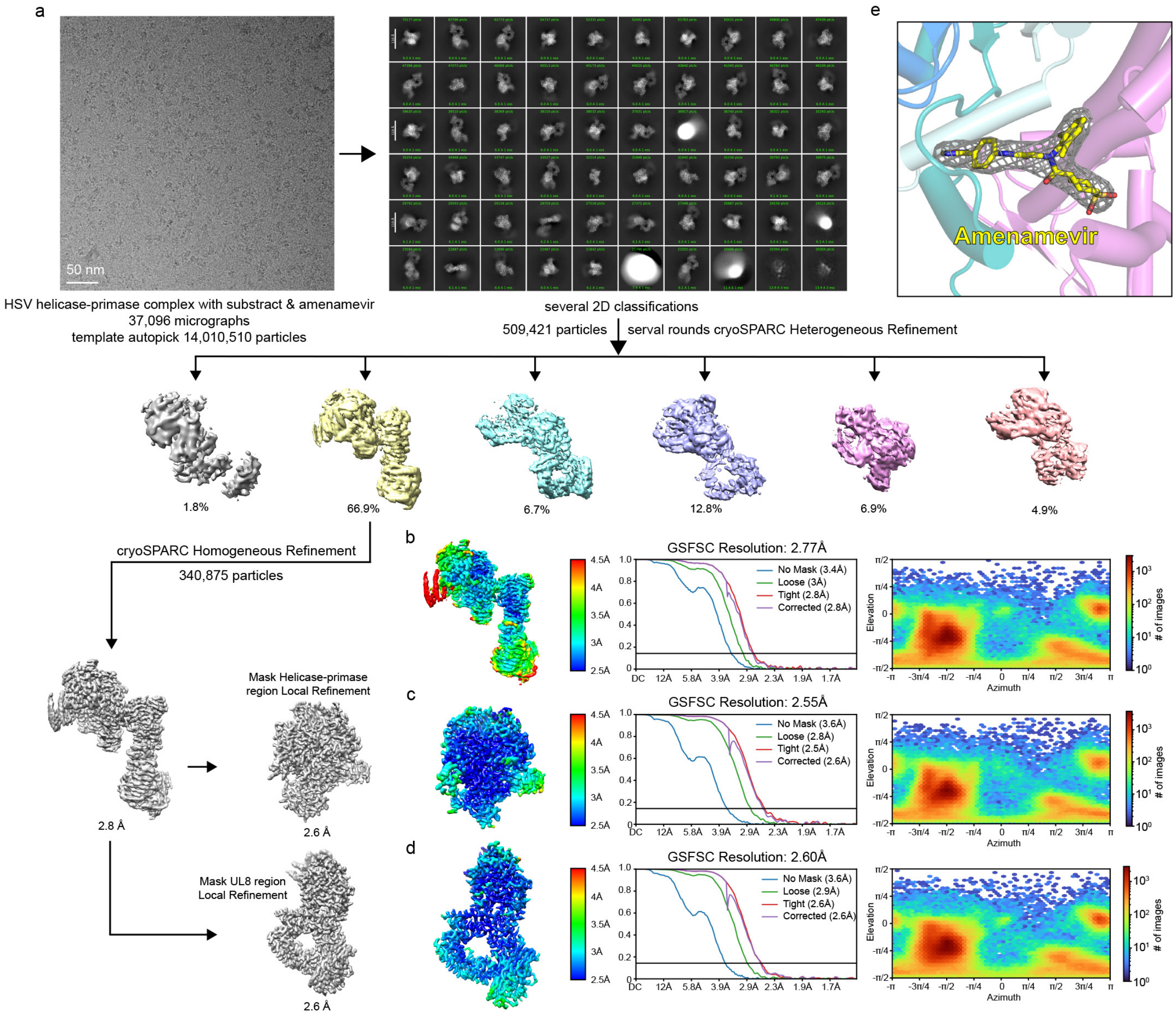

Figure S5. Structure determination of the amenamevir bound HSV H/P complex.

(a) Workflow used for cryo-EM data processing of amenamevir-bound HSV H/P complex.

(b–d) Local resolution estimation, Fourier shell correlation (FSC) curves, and particle angular distributions of the cryo-EM reconstructions of the amenamevir-bound HSV H/P complex at 2.8 Å resolution (b), the masked helicase–primase region at 2.6 Å resolution (c), and the masked UL8 region at 2.6 Å resolution (d).

(e) Cryo-EM density of amenamevir in the drug binding site of the HSV H/P complex. Amenamevir is shown as sticks.

bioRxiv preprint doi: https://doi.org/10.64898/2025.12.23.696259; this version posted December 24, 2025. The copyright holder for this preprint (which was not certified by peer review) is the author/funder, who has granted bioRxiv a license to display the preprint in perpetuity. It is made available under aCC-BY 4.0 International license.

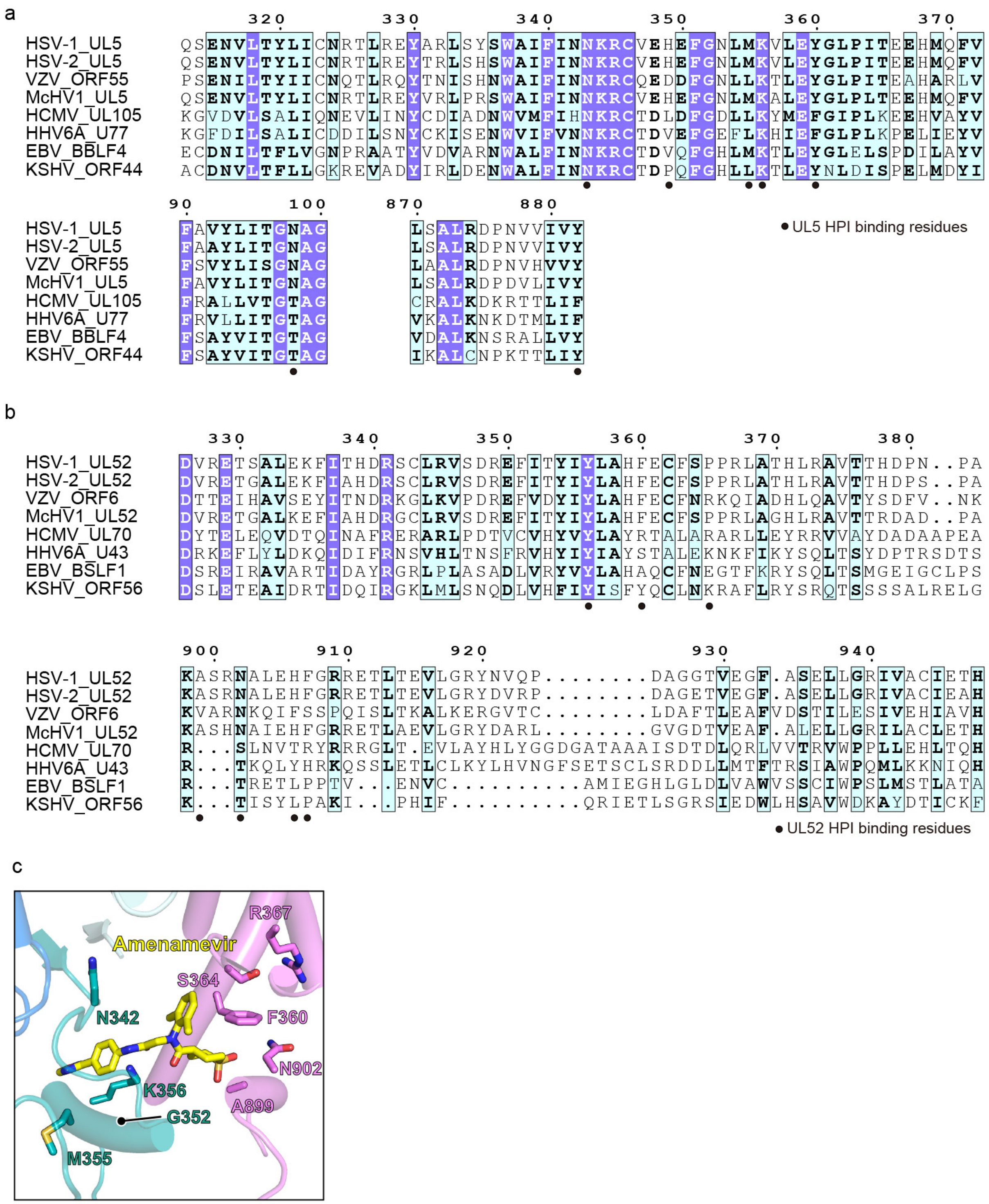

Figure S6. Sequence alignment showing UL5 and UL52 residues that interact with HPIs.

(a and b) Sequence alignments of UL5 (a) or UL52 (b) regions that contain residues interacting with HPIs. These residues are indicated with circles. HSV-1, herpes simplex virus 1, herpes simplex virus 2; VZV, varicella-zoster virus; McHV1, macacine alphaherpesvirus 1; HCMV, human

bioRxiv preprint doi: https://doi.org/10.64898/2025.12.23.696259; this version posted December 24, 2025. The copyright holder for this preprint (which was not certified by peer review) is the author/funder, who has granted bioRxiv a license to display the preprint in perpetuity. It is made available under aCC-BY 4.0 International license.

cytomegalovirus; EBV, Epstein–Barr virus; HHV-6A, human herpesvirus 6A; KSHV, Kaposi's sarcoma-associated herpesvirus.

(c) Close-up view of the amenamevir binding site. Residues that are sites of HPI resistance mutations are shown. See Table S5 for additional information.

bioRxiv preprint doi: https://doi.org/10.64898/2025.12.23.696259; this version posted December 24, 2025. The copyright holder for this preprint (which was not certified by peer review) is the author/funder, who has granted bioRxiv a license to display the preprint in perpetuity. It is made available under aCC-BY 4.0 International license.

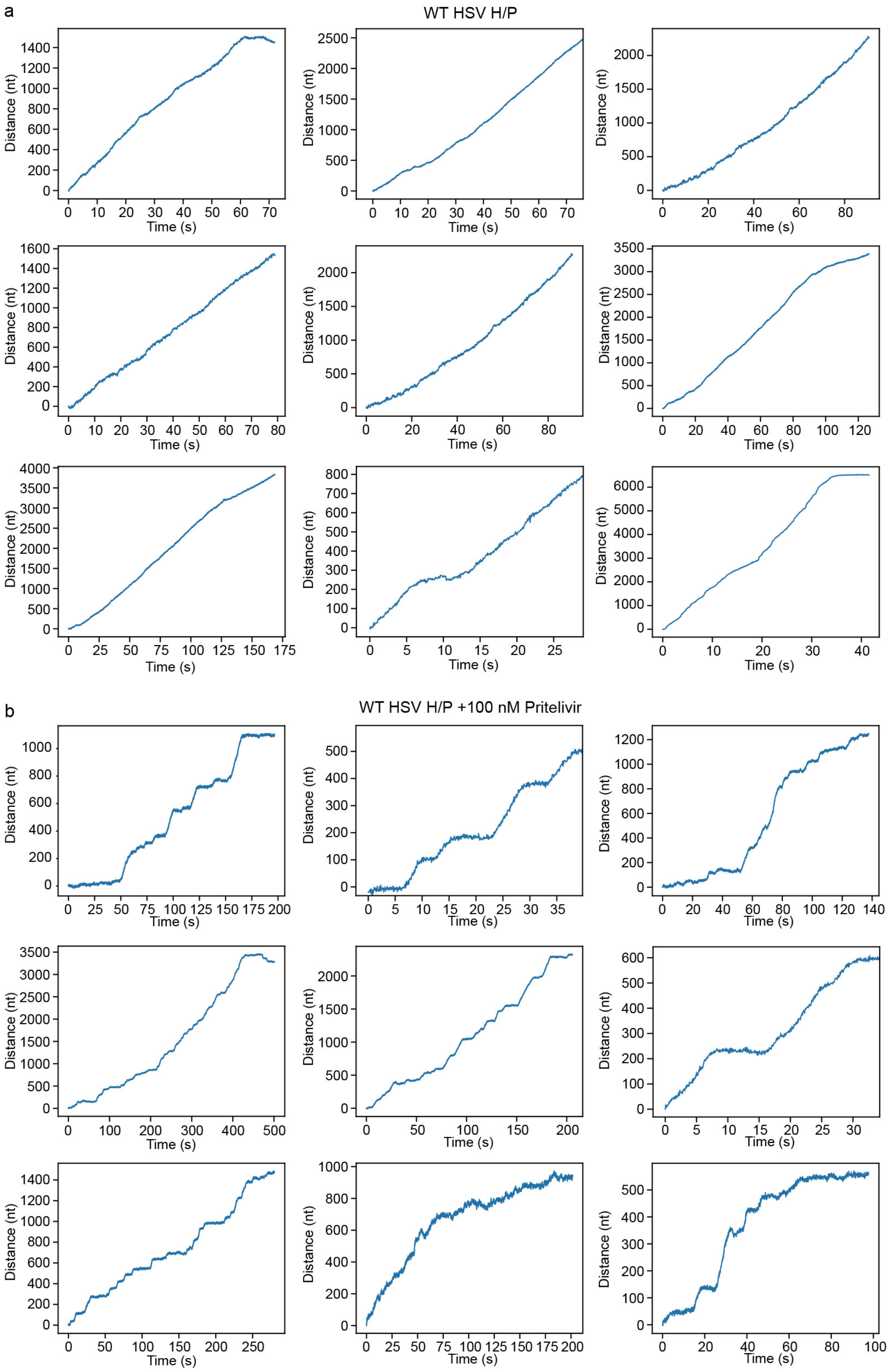

bioRxiv preprint doi: https://doi.org/10.64898/2025.12.23.696259; this version posted December 24, 2025. The copyright holder for this preprint (which was not certified by peer review) is the author/funder, who has granted bioRxiv a license to display the preprint in perpetuity. It is made available under aCC-BY 4.0 International license.

Figure S7. Representative optical tweezer traces of WT HSV H/P complex with and without pritelivir.

(a) Traces of unwinding activity in the presence of 100 nM HSV H/P complex measured in optical tweezer experiments. The experiments with 100 nM H/P complex alone were repeated 16 times.

(b) Traces of unwinding activity in the presence of 100 nM HSV H/P complex and 100 nM pritelivir measured in optical tweezer experiments. The experiments with H/P complex and 100 nM pritelivir were repeated 11 times.

bioRxiv preprint doi: https://doi.org/10.64898/2025.12.23.696259; this version posted December 24, 2025. The copyright holder for this preprint (which was not certified by peer review) is the author/funder, who has granted bioRxiv a license to display the preprint in perpetuity. It is made available under aCC-BY 4.0 International license.

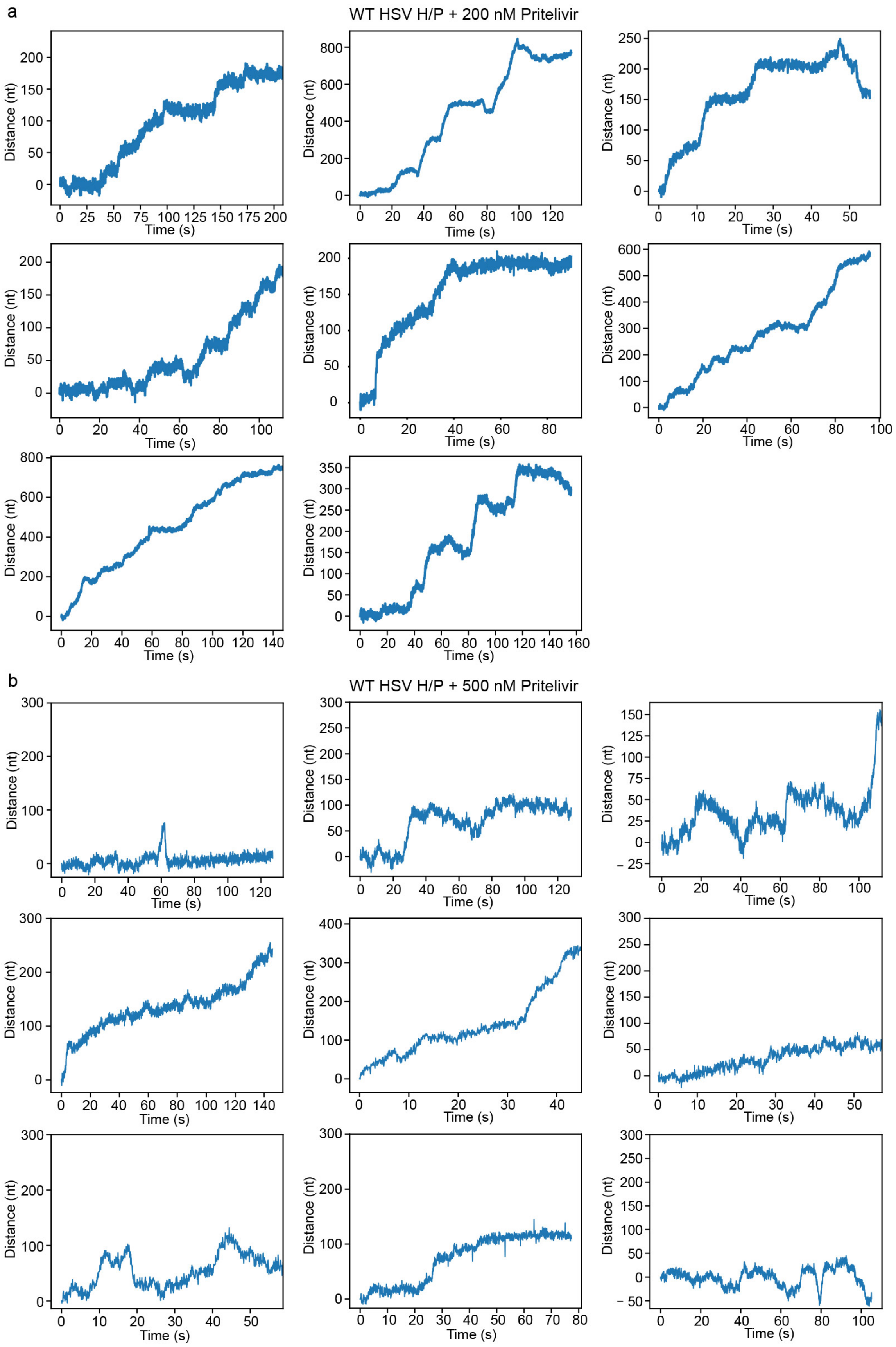

bioRxiv preprint doi: https://doi.org/10.64898/2025.12.23.696259; this version posted December 24, 2025. The copyright holder for this preprint (which was not certified by peer review) is the author/funder, who has granted bioRxiv a license to display the preprint in perpetuity. It is made available under aCC-BY 4.0 International license.

Figure S8. Representative optical tweezer traces of WT HSV H/P complex with pritelivir.

(a) Traces of unwinding activity in the presence of 100 nM HSV H/P complex and 200 nM pritelivir measured in optical tweezer experiments. The experiments with H/P complex and 200 nM pritelivir were repeated 18 times.

(b) Traces of unwinding activity in the presence of 100 nM HSV H/P complex and 500 nM pritelivir measured in optical tweezer experiments. The experiments of H/P complex with 500 nM pritelivir were repeated 12 times.

bioRxiv preprint doi: https://doi.org/10.64898/2025.12.23.696259; this version posted December 24, 2025. The copyright holder for this preprint (which was not certified by peer review) is the author/funder, who has granted bioRxiv a license to display the preprint in perpetuity. It is made available under aCC-BY 4.0 International license.

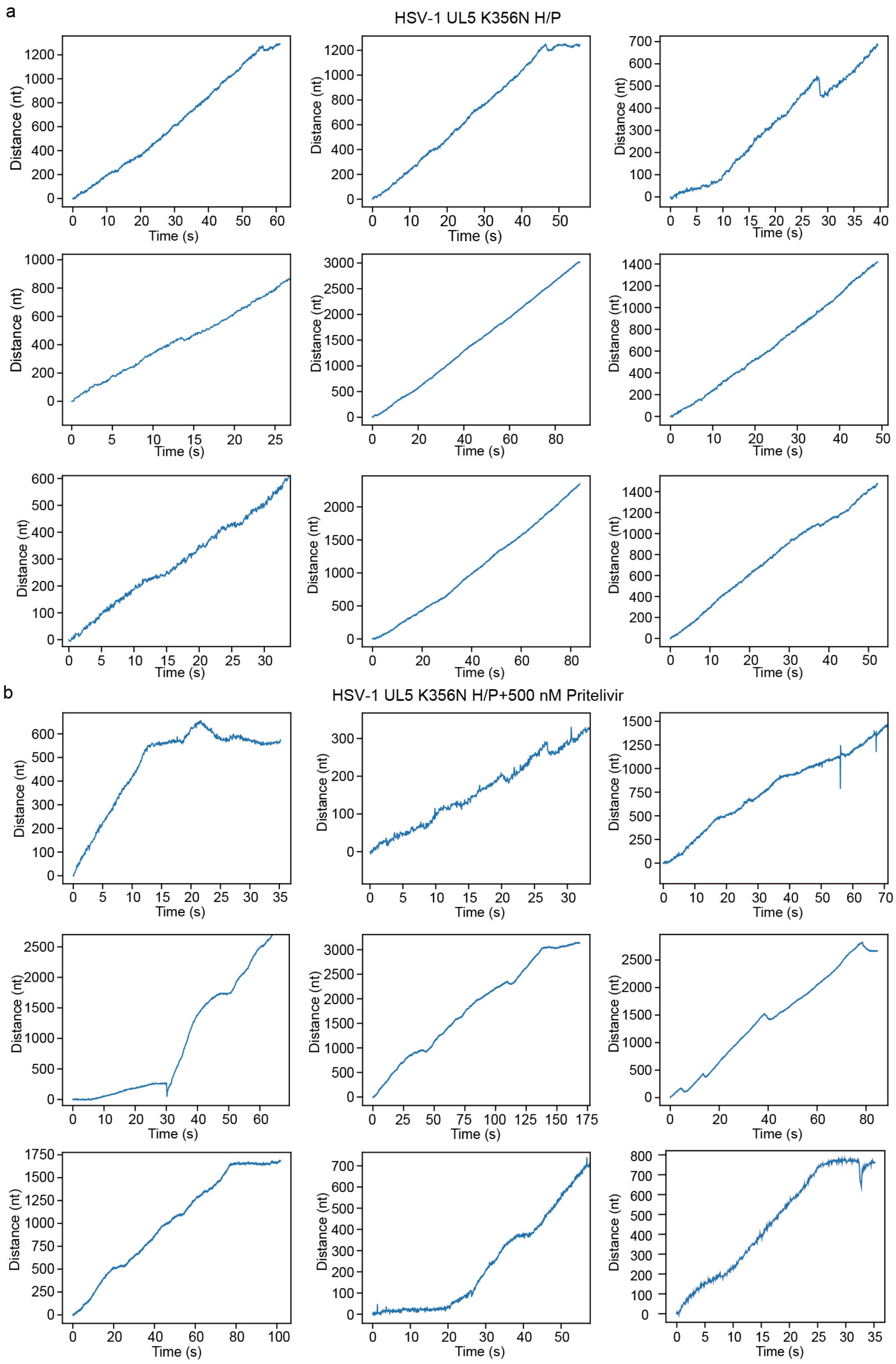

bioRxiv preprint doi: https://doi.org/10.64898/2025.12.23.696259; this version posted December 24, 2025. The copyright holder for this preprint (which was not certified by peer review) is the author/funder, who has granted bioRxiv a license to display the preprint in perpetuity. It is made available under aCC-BY 4.0 International license.

Figure S9. Representative optical tweezer traces of HSV UL5 K356N H/P complex with and without pritelivir.

(a) Traces of unwinding activity in the presence of 100 nM HSV UL5 K356N H/P complex measured in optical tweezer experiments.

(b) Traces of unwinding activity in the presence of 100 nM HSV UL5 K356N H/P complex and 500 nM pritelivir measured in optical tweezer experiments. The experiments of H/P complex with UL5 K356N with or without 500 nM pritelivir were repeated 15 and 17 times, respectively.

bioRxiv preprint doi: https://doi.org/10.64898/2025.12.23.696259; this version posted December 24, 2025. The copyright holder for this preprint (which was not certified by peer review) is the author/funder, who has granted bioRxiv a license to display the preprint in perpetuity. It is made available under aCC-BY 4.0 International license.

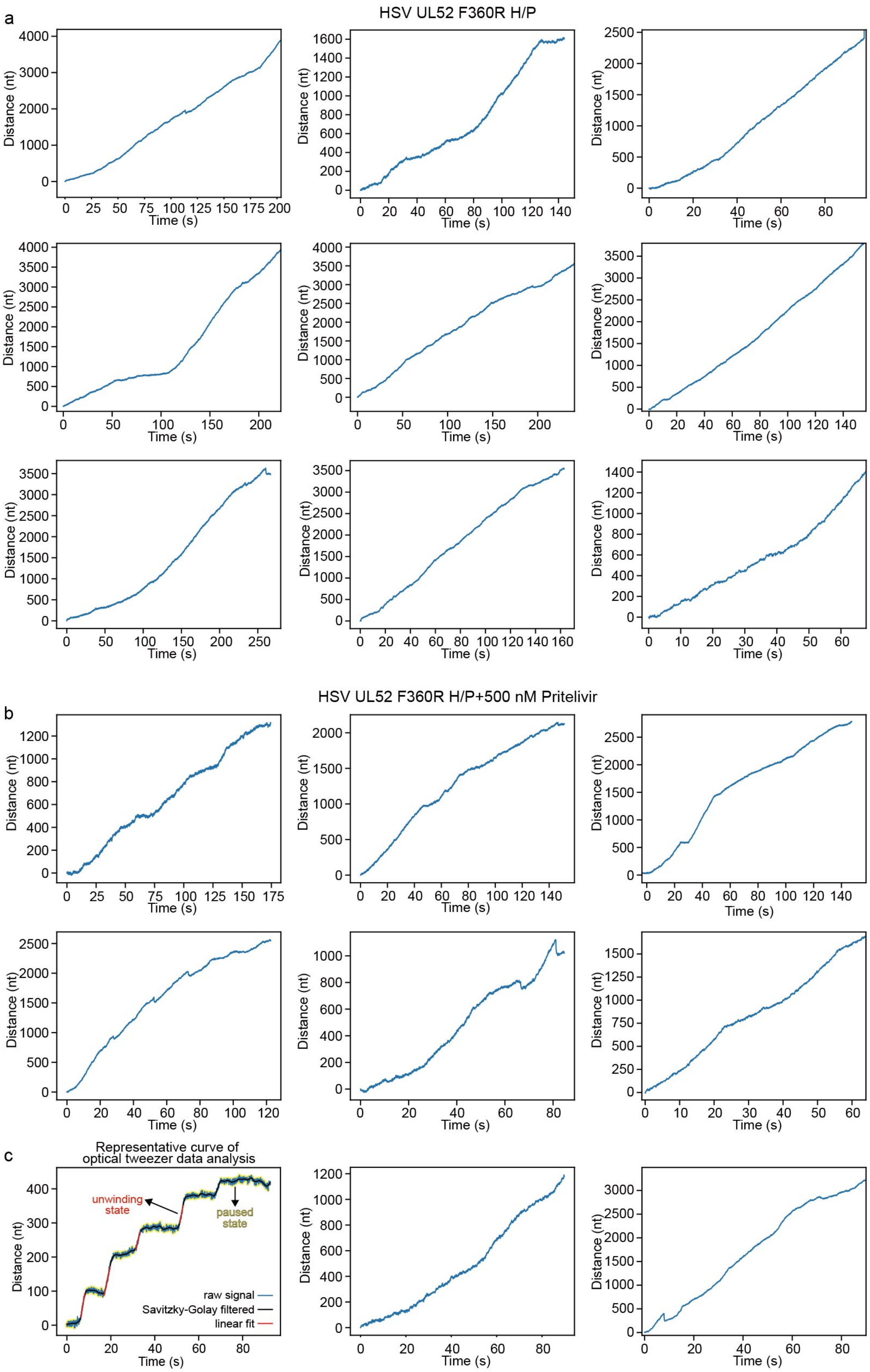

bioRxiv preprint doi: https://doi.org/10.64898/2025.12.23.696259; this version posted December 24, 2025. The copyright holder for this preprint (which was not certified by peer review) is the author/funder, who has granted bioRxiv a license to display the preprint in perpetuity. It is made available under aCC-BY 4.0 International license.

Figure S10. Representative optical tweezer traces of HSV UL52 F360R H/P complex with and without pritelivir.

(a) Traces of unwinding activity in the presence of 100 nM HSV UL52 F360R H/P complex measured in optical tweezer experiments.

(b) Traces of unwinding activity in the presence of 100 nM HSV UL52 F360R H/P complex and 500 nM pritelivir measured in optical tweezer experiments. The experiments of H/P complex with UL52 F360R with or without 500 nM pritelivir were repeated for 11 and 11 times, respectively.

(c) Representative curve of optical tweezer data analysis. This curve is from WT HSV H/P complex with 200 nM pritelivir. The paused state and the unwinding state are labeled after classification based on instantaneous unwinding rate (see Methods).

bioRxiv preprint doi: https://doi.org/10.64898/2025.12.23.696259; this version posted December 24, 2025. The copyright holder for this preprint (which was not certified by peer review) is the author/funder, who has granted bioRxiv a license to display the preprint in perpetuity. It is made available under aCC-BY 4.0 International license.

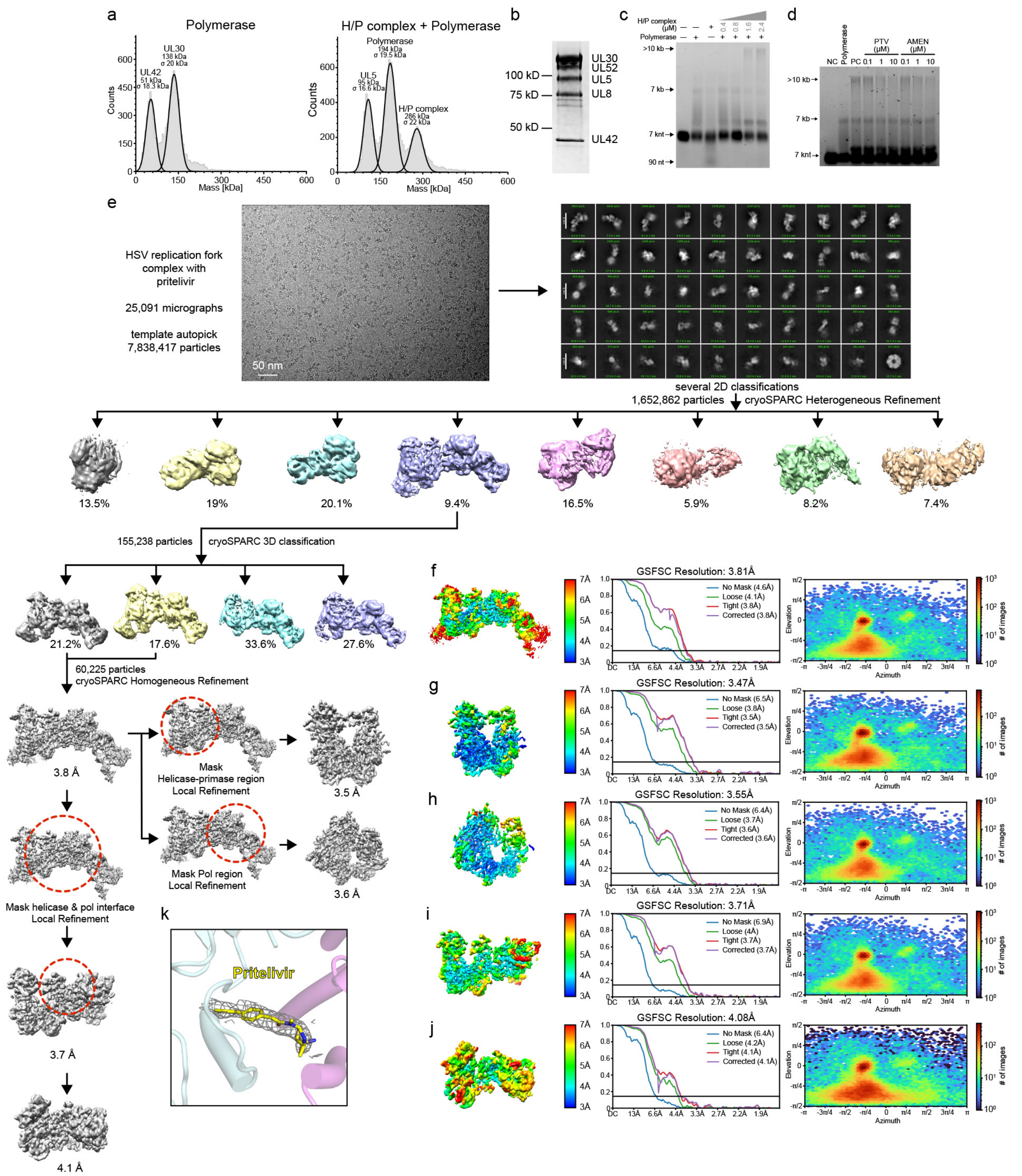

Figure S11. Mass photometry results, rolling-circle assay gels, and structure determination of the pritelivir-bound HSV replication fork complex.

(a) Mass photometry analysis of the HSV polymerase holoenzyme (UL30/UL42) alone or the HSV replication fork complex components (UL5/UL52/UL8+ UL30/UL42) without DNA. The experiment was performed twice, and representative data are shown.

bioRxiv preprint doi: https://doi.org/10.64898/2025.12.23.696259; this version posted December 24, 2025. The copyright holder for this preprint (which was not certified by peer review) is the author/funder, who has granted bioRxiv a license to display the preprint in perpetuity. It is made available under aCC-BY 4.0 International license.

(b) SDS–PAGE analysis of the HSV replication fork complex after size-exclusion chromatography, visualized using a stain-free gel system.

(c) Products of the rolling circle assay performed with HSV polymerase with or without co-incubation with the H/P complex visualized using alkaline agarose gel electrophoresis. This experiment was performed three times, and a representative gel is shown.

(d) Rolling-circle assay performed with the HSV replication fork complex co-incubated with different concentrations of pritelivir (PTV) or amenamevir (AMEN), visualized using alkaline agarose gel electrophoresis. This experiment was performed three times, and a representative gel is shown.

(e) Workflow used for cryo-EM data processing of the HSV replication fork complex.

(f–j) Local resolution estimation, Fourier shell correlation (FSC) curves, and particle angular distributions of the cryo-EM reconstructions of the overall HSV replication fork complex at 3.8 Å resolution (f), masked HSV helicase at 3.5 Å resolution (g), masked HSV polymerase at 3.6 Å resolution (h), masked interface between polymerase and helicase–primase complex at 3.7 Å resolution (i) and masked FYNPYL motif binding site at 4.1 Å resolution (j).

(k) Cryo-EM density of pritelivir in the drug binding site in the HSV replication fork complex structure. Pritelivir is shown as sticks.

bioRxiv preprint doi: https://doi.org/10.64898/2025.12.23.696259; this version posted December 24, 2025. The copyright holder for this preprint (which was not certified by peer review) is the author/funder, who has granted bioRxiv a license to display the preprint in perpetuity. It is made available under aCC-BY 4.0 International license.

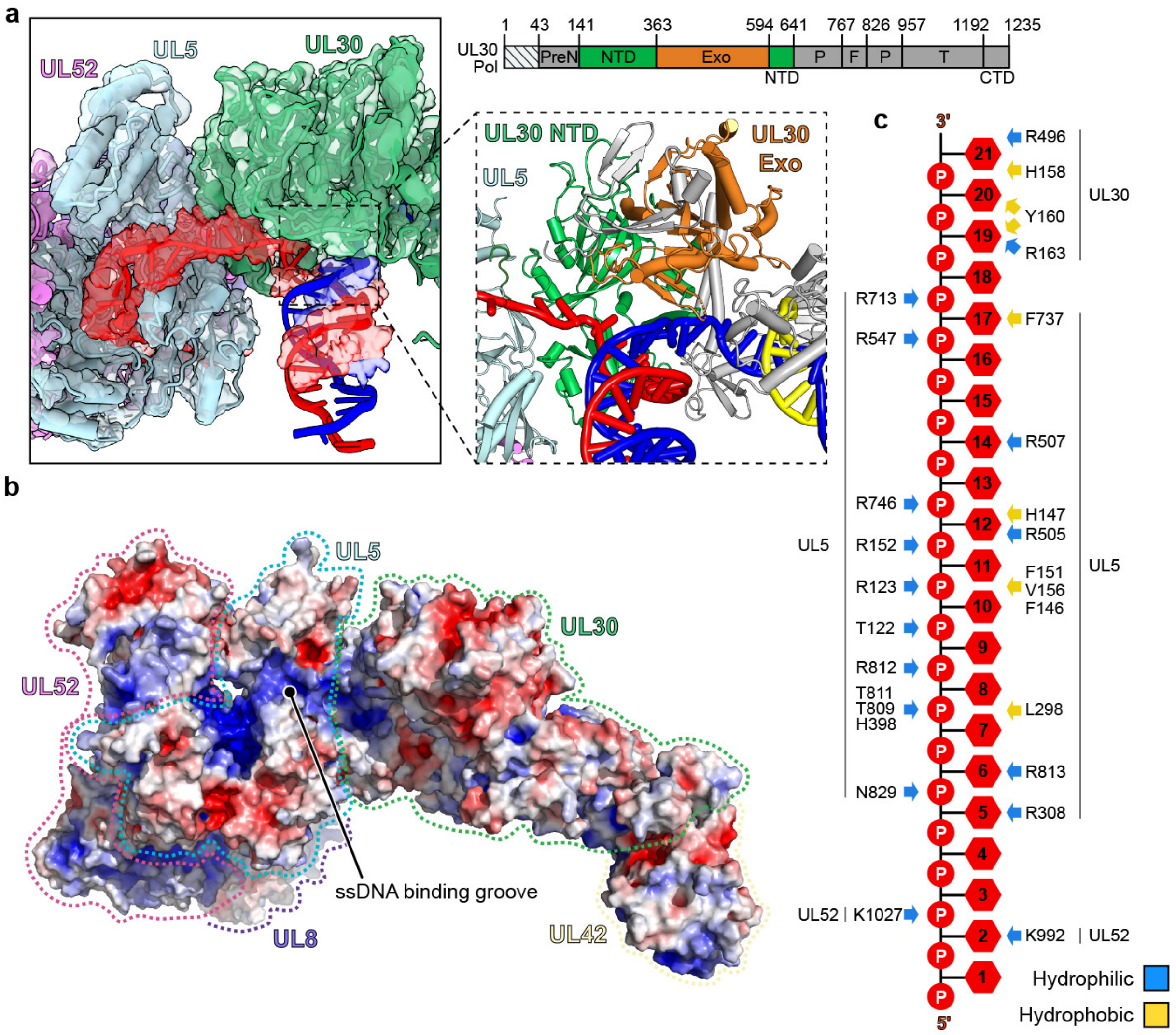

Figure S12. HSV replication fork complex ssDNA and parental dsDNA interactions.

(a) Cryo-EM density (left) of parental DNA in the HSV replication fork complex shown as a surface. The presumed lagging strand is colored red. The presumed leading strand is colored blue. Close-up view (right) of the location of parental DNA. The UL30 NTD shown in green. The UL30 Exo domain is shown in orange. The remaining domains in UL30 are shown in light gray.

(b) Electrostatic surface potential representation of the HSV replication fork complex ssDNA binding groove. Each subunit is labeled.

(c) Schematic representation of the HSV replication fork complex interactions with the presumed lagging strand DNA. Hydrophilic interactions are shown in blue. Hydrophobic interactions are shown in yellow.

bioRxiv preprint doi: https://doi.org/10.64898/2025.12.23.696259; this version posted December 24, 2025. The copyright holder for this preprint (which was not certified by peer review) is the author/funder, who has granted bioRxiv a license to display the preprint in perpetuity. It is made available under aCC-BY 4.0 International license.

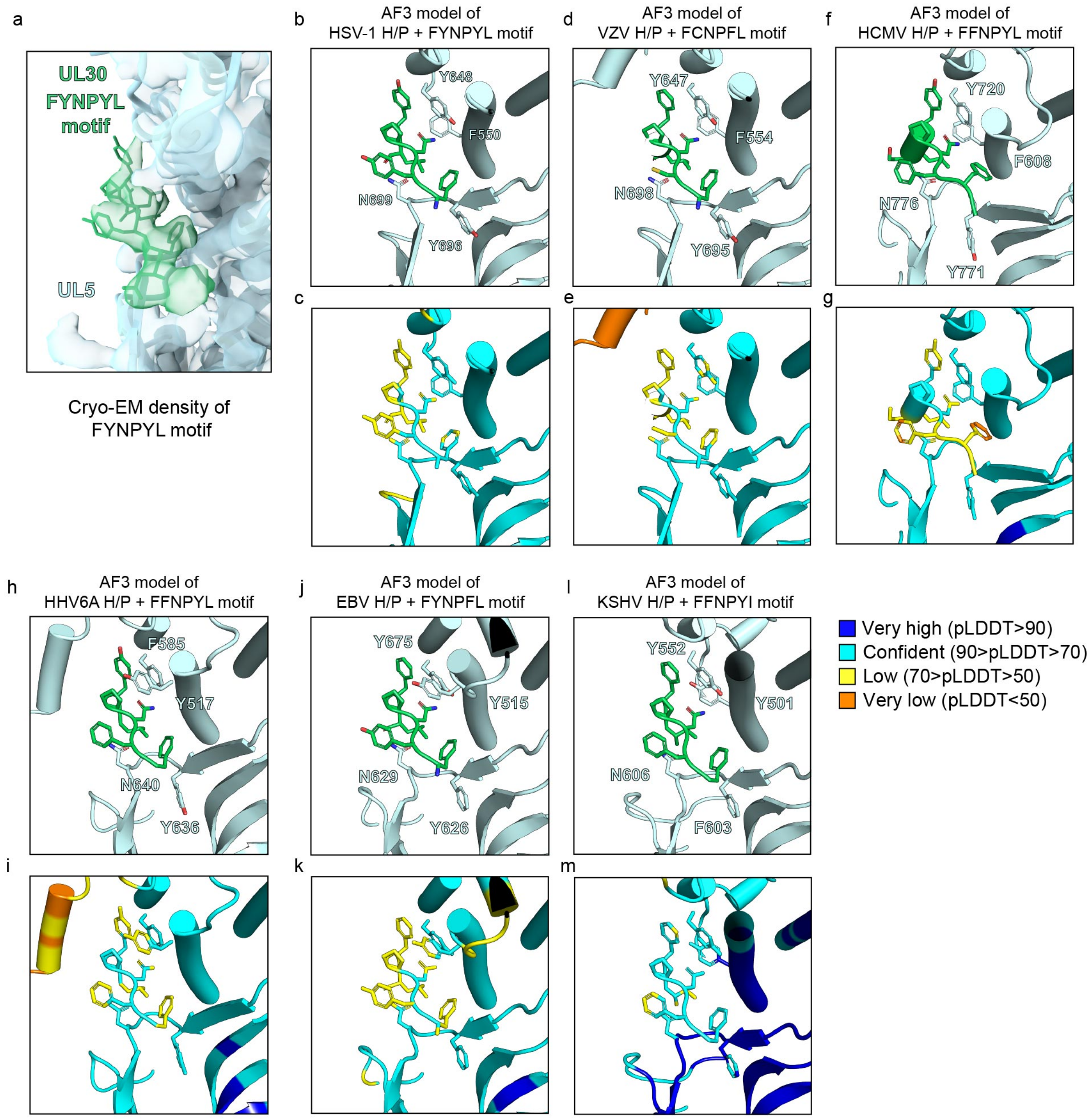

Figure S13. AF3 models of herpesvirus H/P complex bound to polymerase FYNPYL motif.

(a) Cryo-EM density of the UL30 FYNPYL motif in the HSV replication fork complex shown as surface. The UL30 FYNPYL motif is shown as sticks

(b–m) Structures and pLDDT scores of AF3 models of herpesvirus H/P complexes bound to the polymerase preN domain containing the indicated motifs: FYNPYL (HSV, b and c); FCNPFL (VZV, d and e); FFNPYL (HCMV, f and g); FFNPYL (HHV-6A, h and i); FYNPFL (EBV, j and k); and FFNPYI (KSHV, l and m).

bioRxiv preprint doi: https://doi.org/10.64898/2025.12.23.696259; this version posted December 24, 2025. The copyright holder for this preprint (which was not certified by peer review) is the author/funder, who has granted bioRxiv a license to display the preprint in perpetuity. It is made available under aCC-BY 4.0 International license.

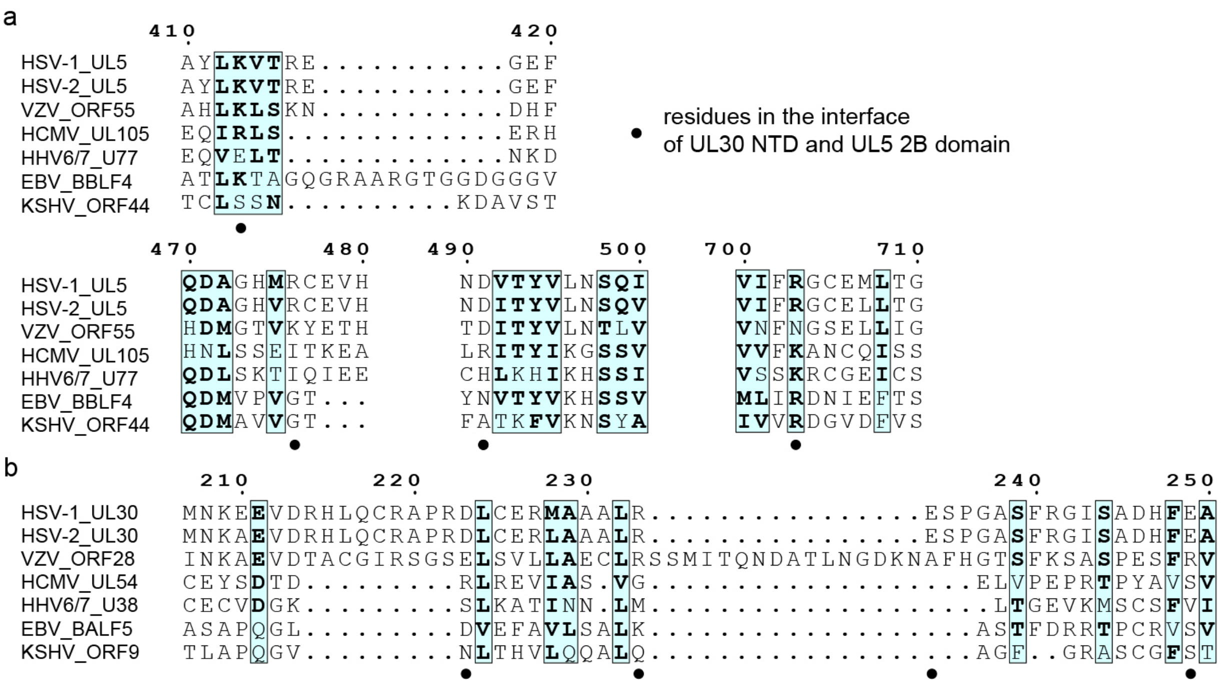

Figure S14. Sequence alignment of interacting residues at the UL5 2B–UL30 NTD interface.

(a and b) Sequence alignment of the HSV UL5 2B domain (a) or the UL30 NTD (b) with the analogous domains of other herpesviruses. Residues involved in interactions at the interface of these domains are indicated. HSV-1, herpes simplex virus 1; HSV-2, herpes simplex virus 2; VZV, varicella-zoster virus; HCMV, human cytomegalovirus; EBV, Epstein–Barr virus; HHV-6A, human herpesvirus 6A; KSHV: Kaposi's sarcoma-associated herpesvirus.

bioRxiv preprint doi: https://doi.org/10.64898/2025.12.23.696259; this version posted December 24, 2025. The copyright holder for this preprint (which was not certified by peer review) is the author/funder, who has granted bioRxiv a license to display the preprint in perpetuity. It is made available under aCC-BY 4.0 International license.

Table S1. Expected masses of subunits and complexes and mass photometry results.

Complex	Subunit mass	Complex mass	Measured mass
Helicase–primase (UL5/UL52/UL8)	99 kDa (UL5)<br>114 kDa (UL52)<br>80 kDa (UL8)	293 kDa	292 s.d. of ± 20 kDa
Polymerase holoenzyme (UL30/UL42)	131 kDa (UL30)<br>35 kDa (UL42)	166 kDa	196 s.d. of ± 33 kDa
Replication fork complex (RFC) with DNA	460 kDa (RFC)<br>30 kDa (DNA)	490 kDa	494 s.d. of ± 52 kDa

Table S2. DNA substrates for cryo-EM and rolling-circle assays.

DNA	Sequence
Fork DNA template	5′-TCC CGC CCG TTT TTT TTT TTT TTT TTT TTT TTT TTT TTT TCG GAG TCG TTT CGA CTC CGA TTT TTA CAC GCT ATG TCG TCA AGT TGT ACC-3′
Fork DNA primer	5′-GGT ACA ACT TGA CGA CAT AGC GTG-3′
M13mp18 primer	5′-TTTTTTTTTTTTTTTTTTTTTTTTTTTTTTTTTTTTTTTTTTTTTTT TTTTTTTTTTTTTTTTTCCCAGTCACGACGTTGTAAAACGACGGCCAGT-3′

Table S3. Cryo-EM data collection and image processing for drug-bound H/P complex structures, related to Figures 1 and S1.

	HSV H/P complex bound to pritelivir			HSV H/P complex bound to IM-250		HSV H/P complex bound to amenamevir		
Magnification	105,000			165,000		165,000		
Voltage (kV)	300			300		300		
Pixel Size (Å)	0.83			0.73		0.73		
Electron dose (e/Å^2)	50			52		51		
Defocus range (μm)	-1.0 to -2.5			-1.0 to -2.5		-1.0 to -2.5		
Initial particles	9,118,480			5,535,965		14,010,510		
Final particles	64,227			177,199		340,875		
Symmetry imposed	C1			C1		C1		
FSC threshold	0.143			0.143		0.143		
Maps	Overall map	UL5-UL52 interface	UL52-UL8 interface	Overall map	UL5-UL52 interface	Overall map	UL5-UL52 interface	UL8-UL52 interface
Map resolution (Å)	3.7	3.4	3.3	2.9	3.2	2.8	2.6	2.6
**Model refinement and validation**								
Initial model used	AF3 model							

bioRxiv preprint doi: https://doi.org/10.64898/2025.12.23.696259; this version posted December 24, 2025. The copyright holder for this preprint (which was not certified by peer review) is the author/funder, who has granted bioRxiv a license to display the preprint in perpetuity. It is made available under aCC-BY 4.0 International license.

R.m.s deviations			
Bonds lengths (Å)	0.007	0.008	0.008
Bonds angles (º)	1.107	1.157	1.319
Validation			
Clashscore	11	11	12
Favored (%)	94.2	95.0	94.5
Allowed (%)	5.8	5.0	5.5
Disallowed (%)	0	0	0

	HSV replication fork complex				
Magnification	105,000				
Voltage (kV)	300				
Pixel Size (Å)	0.83				
Electron dose (e/Å^2)	51				
Defocus range (µm)	-1.0 to -2.5				
Initial particles	7,838,417				
Final particles	60,225				
Symmetry imposed	C1				
FSC threshold	0.143				
Maps	Overall map	Helicase UL5	Polymerase UL30	Helicase-polymerase inteface	Masked FYNPYL motif density
Map resolution (Å)	3.8	3.5	3.6	3.7	4.1
**Model refinement and validation**					
Initial model used	PDB: 2GV9 & PDB: 1DML & PDB: 8V1Q				
R.m.s deviations					
Bonds lengths (Å)	0.015				

bioRxiv preprint doi: https://doi.org/10.64898/2025.12.23.696259; this version posted December 24, 2025. The copyright holder for this preprint (which was not certified by peer review) is the author/funder, who has granted bioRxiv a license to display the preprint in perpetuity. It is made available under aCC-BY 4.0 International license.

Bonds angles (º)	1.441
Validation	
Clashscore	12
Favored (%)	95.8
Allowed (%)	4.2
Disallowed (%)	0

Table S4. Summary of previously published mutational analyses.

Subunit	Mutant	Location	Effects	Reference
UL52	C1023A C1028A	ZnF zinc-coordinating residues	Near complete loss of helicase and primase activity of purified UL5–UL52 subcomplex. Mutant UL52 could not complement UL52-null virus, indicating loss of essential function.	Biswas et al.[43] Chen et al.[44]
UL52	D628A D630A	Primase active site residues involved in metal coordination	Decrease primase activity, no effect on helicase activity.	Dracheva et al.[13] Klinedinst et al.[42]
UL5	D249A E250A	ATPase active site residues involved in metal coordination	Mutations profoundly decrease DNA-dependent ATPase activity. Substitutions abolish DNA replication in cells.	Zhu et al.[10] Graves-Woodward et al.[41]
UL5	K103A R345K	ATPase active site residues involved in phosphate coordination	Substitutions of residues abolish DNA replication in cells.	Zhu et al.[10]
UL30	FYNPYL motif mutant to poly A_6	preN motif residues interacting with UL5	No effects on polymerase DNA synthesis activity but impairs viral replication in cells. Severely impairs replication in sensory ganglia and latency establishment.	Terrell et al.[18] Terrell et al.[19]

Table S5. Summary of previously published drug-resistant mutation profiles.

Subunit	Drug	Drug resistance	Location	Effects	Reference
UL5	Pritelivir Amenamevir	N342K	UL5 2A domain	Re-organization of pocket residues	Biswas et al.[50] Sato et al.[53]

bioRxiv preprint doi: https://doi.org/10.64898/2025.12.23.696259; this version posted December 24, 2025. The copyright holder for this preprint (which was not certified by peer review) is the author/funder, who has granted bioRxiv a license to display the preprint in perpetuity. It is made available under aCC-BY 4.0 International license.

UL5	Pritelivir<br>Amenamevir<br>IM-250	G352C<br>G352V<br>G352R	UL5 2A domain	Cause steric hindrance	Biswas et al.[50]<br>Biswas et al.[55]<br>Biswas et al.[48]<br>Gege et al.[33]<br>James et al.[52]<br>Sato et al.[53]
UL5	Pritelivir<br>Amenamevir<br>IM-250	M355I<br>M355T	UL5 2A domain	Modifies HPI contact residue	Biswas et al.[50]<br>Gege et al.[33]<br>James et al.[52]<br>Sato et al.[53]
UL5	Pritelivir<br>Amenamevir<br>IM-250	K356N<br>K356T<br>K356Q	UL5 2A domain	Modifies HPI contact residue	Biswas et al.[50]<br>Biswas et al.[55]<br>Biswas et al.[48]<br>Biswas et al.[49]<br>Gege et al.[33]<br>James et al.[52]<br>Sato et al.[53]<br>Schalkwijk et al.[54]<br>Sulka et al.[51]
UL52	Pritelivir<br>Amenamevir	F360C/V	UL52 NTD	Modifies HPI contact residue	Sato et al.[53]
UL52	Amenamevir	S364G	UL52 NTD	Situated near residues that make direct interactions with amenamevir's unique 2,6-xylyl group	Shiraki et al.[46]
UL52	Amenamevir	R367H	UL52 NTD	Situated near residues that make direct interactions with amenamevir's unique 2,6-xylyl group	Shiraki et al.[46]
UL52	Pritelivir<br>Amenamevir	A899T	UL52 middle domain	Modifies HPI contact residue	Biswas et al.[50]<br>James et al.[52]<br>Sato et al.[53]<br>Schalkwijk et al.[54]
UL52	Pritelivir<br>Amenamevir	N902T	UL52 middle domain	Modifies HPI contact residue	Sato et al.[53]
